# Examining the role of different age groups, and of vaccination during the 2012 Minnesota pertussis outbreak

**DOI:** 10.1038/srep13182

**Published:** 2015-08-17

**Authors:** Colin J. Worby, Cynthia Kenyon, Ruth Lynfield, Marc Lipsitch, Edward Goldstein

**Affiliations:** 1 Center for Communicable Disease Dynamics, Department of Epidemiology, Harvard T.H. Chan School of Public Health, 677 Huntington Ave., Boston, MA 02115, USA; 2 Minnesota Department of Health, St. Paul, MN, USA; 3 Department of Immunology and Infectious Disease, Harvard T.H. Chan School of Public Health, 677 Huntington Ave., Boston, MA 02115, USA

## Abstract

There is limited information on the roles of different age groups during pertussis outbreaks. Little is known about vaccine effectiveness against pertussis infection (both clinically apparent and subclinical), which is different from effectiveness against reportable pertussis disease, with the former influencing the impact of vaccination on pertussis transmission in the community. For the 2012 pertussis outbreak in Minnesota, we estimated odds ratios for case counts in pairs of population groups before vs. after the epidemic’s peak. We found children aged 11–12y, 13–14y and 8–10y experienced the greatest rates of depletion of susceptible individuals during the outbreak’s ascent, with all ORs for each of those age groups vs. groups outside this age range significantly above 1, with the highest ORs for ages 11–12y. Receipt of the fifth dose of DTaP was associated with a decreased relative role during the outbreak’s ascent compared to non-receipt [OR 0.16 (0.01, 0.84) for children aged 5, 0.13 (0.003, 0.82) for ages 8–10y, indicating a protective effect of DTaP against pertussis infection. No analogous effect of Tdap was detected. Our results suggest that children aged 8–14y played a key role in propagating this outbreak. The impact of immunization with Tdap on pertussis infection requires further investigation.

The resurgence of pertussis (whooping cough) in the past two decades has been the cause of much concern, with the number of reported cases in 2012 in the United States being the highest in fifty years^1^. The causes of this resurgence are still not completely understood; in particular, there is uncertainty about the role played by different age groups and the impact of vaccination on the transmission of infection^2^,^3^,^4^.

While pertussis infection in adults and adolescents can be mild, and is often undiagnosed, morbidity and mortality in young children, particularly infants is significantly higher^2^. Adults and adolescents are thought to be an important source of infection for infants^5^, but the relative role of different age groups in transmission during the course of an outbreak remains unclear. While surveillance data provide counts for the numbers of reported cases of pertussis disease in different age groups, those counts may not serve as a reliable indicator of the incidence rates for pertussis infection (both clinically apparent and subclinical infection) since the reporting rates for cases of pertussis infection vary by age group due to differences in the severity of disease and disease reporting practices. Moreover, it can happen that groups with the highest rates of reported cases are not the groups with the highest incidence rates of infection^6^. Altogether, there is a good deal of uncertainty about the relative magnitude of the incidence rates of pertussis infection in different age groups with the corresponding ambiguity about those groups’ relative role in pertussis transmission in the community.

Vaccination is one of the key control measures employed to protect a community from pertussis disease. Acellular pertussis vaccines were introduced in the 1990s. In the US, children receive five diphtheria, tetanus and acellular pertussis (DTaP) vaccines before the age of 7 years. In 2005, a booster vaccine (tetanus, reduced diphtheria, and reduced acellular pertussis (Tdap)) was introduced and recommended at age 11–12 years in response to an increasing number of reported cases in adolescents.

Over the past ten years, reported cases of pertussis have increased considerably in children and adolescents^7^, generating debate as to whether these vaccines should be altered or replaced^8^. Several studies have demonstrated the protection provided by acellular pertussis vaccines against symptomatic pertussis episodes in different age cohorts, but studies have also shown the earlier than expected waning of vaccine efficacy^9^,^10^,^11^,^12^,^13^, particularly when compared to the whole cell vaccine^14^,^15^. A different type of immune response for the acellular vaccine compared with the whole cell vaccine likely plays a role in the increase in reported cases^16^. At the same time, little is known about vaccine effectiveness against infection with *Bordetella pertussis* and onward transmission. That effectiveness may differ from protection against clinical or symptomatic disease^17^; indeed, a recent study found that infant baboons vaccinated with acellular pertussis vaccine can be asymptomatically infected and transmit onward^3^. Given that only a small proportion of pertussis infections are reported^6^,^18^,^19^, it is important to explore the effectiveness against development of infection to determine the impact of vaccination on pertussis transmission in the community.

In this paper we introduce a method to estimate the relative importance of different age groups in the transmission of pertussis infection and the impact of vaccination on protection from infection with *Bordetella pertussis*. This method is based on our recent analogous work in the context of influenza^20^. The approach is to identify groups that are disproportionately represented in incidence during the ascent of the outbreak compared to the descent. The rationale behind this is that groups with increased susceptibility to pertussis infection and/or higher contact rates will experience a disproportionate depletion among their pool of susceptibles early in the outbreak, and thus have a decreasing share of all cases as the epidemic progresses.

While we don’t have information on the rates of pertussis infection, with reported cases constituting only a small fraction of all incident cases of infection^6^,^18^,^19^, our approach only requires gauging the relative changes in the proportions of different groups in the overall incidence of infections through time. This can be achieved using data on reported pertussis cases, provided that case-reporting ratios (ratios of the number of reported cases to the number of incident cases) within each group are constant throughout the outbreak. In particular, our method allows for variability in case reporting rates across different groups.

In this paper we apply the above approach to data from the 2012 pertussis outbreak in Minnesota. We assessed the relative change in incidence during the outbreak’s ascent vs. descent periods for the different age groups, in order to identify age groups that had a disproportionate relative role in incidence. Furthermore, we assessed the relative change in incidence among vaccinated vs. under-vaccinated individuals in each age group, to examine a potential protective effect of vaccination against pertussis infection.

## Methods

### Data

We considered data on reported pertussis cases in Minnesota between 2010 and 2013. The pertussis outbreak in 2012 was the largest recorded in nearly seventy years^21^. We used case records collected from 19 counties in Minnesota (see Appendix: Table S1, Figure S1) that experienced large numbers of cases in 2012. Cases reported in these MN counties represented 91% of total cases reported in 2012. Variables of interest were pulled from Minnesota’s routine surveillance system and included: onset date, age (in months), county and vaccination history (if available).

**Figure 1:**
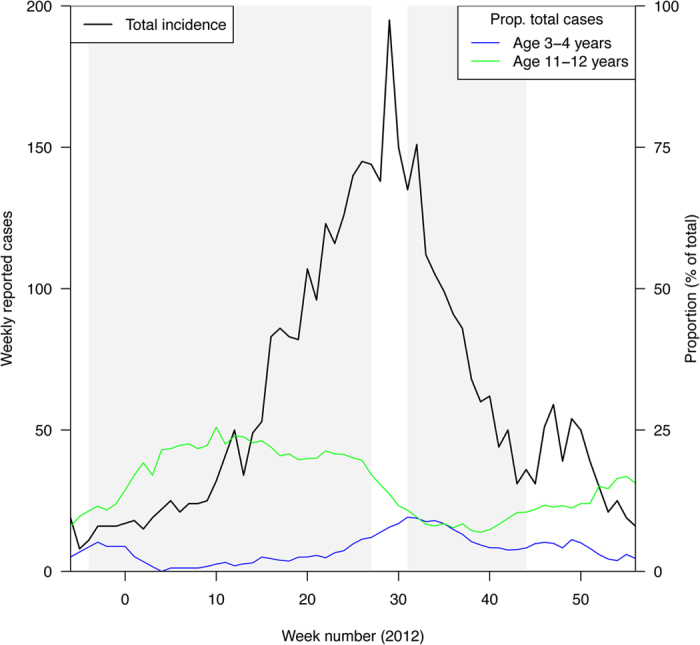
Total weekly number of pertussis cases between week 46, 2011 and week 4, 2013, and the proportions of total cases among children aged 11–12 and 3–4 years. The pre-peak and post-peak periods as defined in the Methods section are shaded in gray. Proportions shown were calculated using a five-week moving average.

We defined the peak week as the calendar week in 2012 with the highest total number of recorded cases, which was week 29. The pre-peak (ascent) period was defined as the period between calendar week 48 in 2011 through calendar week 27 in 2012 (Fig. 1). The post-peak (descent) period was defined as calendar weeks 31 through 42 of 2012, with the latter cut-off chosen due to the emergence of a second, smaller, peak in incidence in 2012 (Fig. 1).

### Age group analysis

Our approach utilizes the odds ratio to readily assess which age group among a pair experiences a larger depletion of susceptible individuals during an outbreak’s ascent. Age was split into 9 groups: (<1, 1–2, 3–4, 5–7, 8–10, 11–12, 13–14, 15–19, 20+ years). For each pair of groups (say *g*_1_ and *g*_2_), we calculated the odds ratio for belonging to either before vs. after the outbreak’s peak, 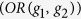.

In order to compare trends among several age groups simultaneously, we also reviewed relative risks. For each age group *g* we calculated the following: the proportion of the cases in age group *g* among all the cases during the pre-peak period, *B*(*g*); the corresponding proportion for the after-the-peak period, *A*(*g*); and the relative risk (RR):


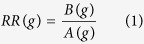


Groups with higher values of *RR*(*g*) experience a larger relative change in the number of cases for the pre-peak vs. post-peak periods, suggesting a larger relative depletion of the pool of susceptibles during the epidemic’s ascent compared to groups with lower values of *RR*(*g*). In fact, relative risks relate to the aforementioned pairwise odds ratios for the different age groups (*g*_1_ and *g*_2_) as follows:


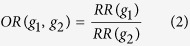


which explains why considering relative risks yields the same pairwise comparison results as by considering the odds ratios. In the Appendix, we demonstrate that calculating the relative risk depends only on a proxy of true incidence (which in our analysis is given by the reported cases of pertussis), and does not require reporting rates to be known, or identical, across groups.

### Vaccination status analysis

We performed separate analyses for vaccination status within different age cohorts. Children with incomplete vaccination history, history of receipt of whole-cell pertussis vaccines or receipt of acellular pertussis vaccine within one month prior to onset of illness were excluded from the analysis. The ACIP recommends a five-dose vaccination schedule for DTaP administered at ages 2, 4, and 6 months, 15–18 months and 4–6 years, while Tdap is recommended for ages 11–12 years (though some vaccination of 10 year olds took place in autumn 2012, when the epidemic was waning). We attempted to select age groups that were approximately homogeneous in terms of vaccine coverage levels. Therefore, for example, the 5–7 year age group was split into the aged 5 and 6–7 year groups as the proportion of cases among the 5 year olds who have not received the 5^th^ dose of DTaP was 26.9%, with those proportions among the 6 and 7 year olds being 6.3% and 4.1% correspondingly.

The vaccination and age groups examined were Tdap for children aged 11–12 years and 13–14 years; 5^th^ dose of DTaP for ages 5, 6–7 and 8–10 years; 4^th^ dose of DTaP for ages 19–47 months and 15–36 months; 3^rd^ dose of DTaP for ages 7–14 months and 6–12 months. We note that overlapping age groups were considered for the 3^rd^ and the 4^th^ doses of DTaP because the relevant age group was too small to split and we sought more than one estimate for the corresponding doses. For each category of children, we considered the age-specific odds ratios for being vaccinated vs. under-vaccinated for the pre-peak vs. post-peak periods, classifying those lacking the dose in question as under-vaccinated. We considered the age-specific odds ratios for being vaccinated vs. under-vaccinated for the pre-peak vs. post-peak periods. An odds ratio below one is interpreted as protective, indicating a smaller depletion of susceptibles among the vaccinated vs. the under-vaccinated children during the ascent period of the epidemic.

### Ethics statement

This research was determined to be exempt by the Institutional Review Boards both at the Harvard School of Public Health (protocol IRB 14–0413) and the Minnesota Department of Health (protocol IRB 14–329).

## Results

### Age-group analysis

**Figure 2:**
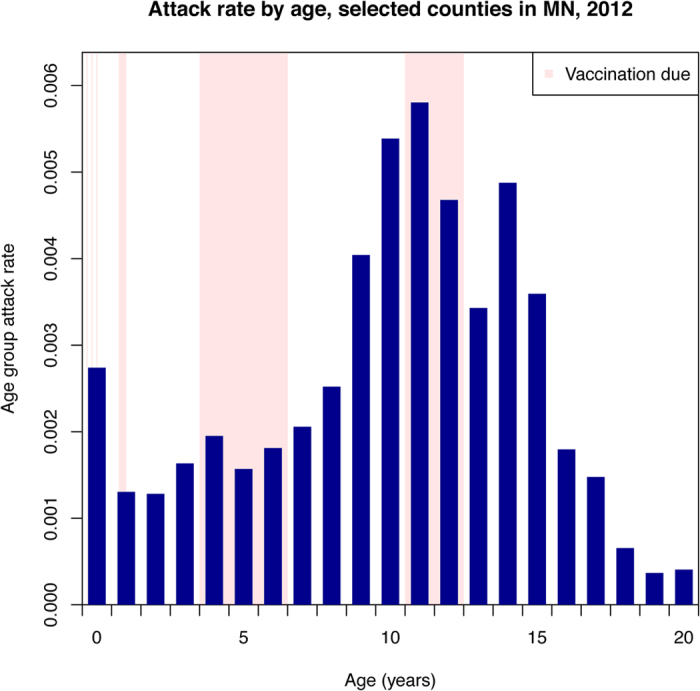
Rates of reported pertussis cases in 2012 by age for the 19 Minnesota counties used in the main analysis.

Figure 2 presents the rates of reported pertussis cases in 2012 for the 19 Minnesota counties used in the main analysis. Rates varied significantly with age, which potentially reflects differences in susceptibility, contact rates, variability in reporting, and the impact of vaccination. Figure 1 shows the total weekly numbers of cases during the outbreak and the (time-changing) proportions of those cases aged between 3–4 and 11–12 years.

Table 1 presents the estimates of the relative risk *RR*(*g*) in the selected age groups. High values of *RR* were estimated for children between 8–14 years of age, with the highest *RR* recorded for 11–12 year olds. Table 2 provides a pairwise comparison of age groups, indicating that 8–14 year olds experienced a significantly greater depletion of susceptibles during the outbreak’s ascent than any other age group, with the depletion of susceptibles among 11–12 year olds significantly higher than among 8–10 year olds.

### Vaccination status analysis

Table 3 records the odds ratios for being vaccinated vs. under-vaccinated for DTaP for the pre- vs. post-peak periods in select age groups. There was a significant difference in the proportion of under-vaccinated cases within both the 5 year and 8–10 year age groups, indicating that under-vaccinated individuals had higher incidence than fully vaccinated individuals during the ascent stage, and suggesting a protective effect against pertussis infection from the 5th dose of DTaP. No significant effect was observed within the 6–7 year age group; however, the number of under-vaccinated cases was very low, leading to a wide confidence interval. Results for younger age groups suggest a protective effect associated with the 3^rd^ and 4^th^ dose of DTaP, however, these did not reach significance.

Table 4 records the results of the corresponding analysis for Tdap. When evaluating Tdap among 11–12 and 13–14 year olds, we found no significant change in proportions pre- and post-peak in either age group. Although confidence intervals were again wide, the results imply a lack of evidence that Tdap confers protection against infection during an outbreak (Table 4).

## Discussion

Our analysis of pertussis case report data highlights the important role played by 8–14 year old children in the beginning of the 2012 outbreak in Minnesota, with a particularly large relative depletion of susceptibles inferred among 11–12 year olds. These results suggest that providing additional protection against pertussis infection for these groups should reduce the risk of large outbreaks (further investigations presented in the Appendix), protecting vulnerable sub-populations, particularly infants.

Our assessment of the relative roles of the different age groups in pertussis outbreaks is based on a new inference method that examines the relative change in the proportion of each group among all reported cases of pertussis during the outbreak’s ascent vs. descent. This method is not influenced by the different case reporting rates in different age groups (see Appendix) and in particular it helps address the ambiguity about the roles of the different age groups that are traditionally evaluated using data on reported cases of pertussis disease. For example, it had been suggested that adults may play an important role in pertussis transmission^22^, even though relatively few cases are reported in adults, reflecting a low case-reporting rate in adults; other studies argue for a limited role of adults in pertussis transmission in the community^23^. Our results on the relative risks in different age groups suggest that at least for the 2012 outbreak in Minnesota, the relative role of a young adolescent was far greater than that of one adult.

The key premise of our approach is that the RR statistic, which is related to depletion of susceptible individuals in different age groups during the outbreak’s ascent reflects the role that those groups play in driving epidemics. This premise is supported by our earlier work on influenza^20^ where we have shown that for the larger epidemics, vaccinating groups with the highest RR would result in the biggest impact on transmission dynamics in the whole community. In the Appendix, we perform analogous analyses in the context of pertussis, reaching similar conclusions.

The introduction of the Tdap booster vaccine in 2005 was intended to protect adolescents from symptomatic infection; however, we have identified these age groups playing a key role in the early stages of the 2012 pertussis outbreak in Minnesota. There is some evidence that Tdap provides moderate protection against symptomatic or reported pertussis episodes in adolescents^9^,^13^,^24^,^25^, and the decline in the rate of reported cases from the age of 11 to 13 years in our dataset (Fig. 2) may reflect this protection. At the same time, there is evidence that efficacy of acellular pertussis vaccines against pertussis infection and further transmission may be different from efficacy against symptomatic infection^3^,^26^. Our results refine that evidence, showing no differences in the Tdap vaccination status of cases aged 11–14 years between the ascent and descent of the outbreak, which is consistent with no protection of Tdap against pertussis infection during an outbreak. This further brings into question the impact of Tdap vaccination campaigns, both among adolescent and adults, on pertussis transmission in the community.

Our results on the Tdap vaccination status of cases during the outbreak’s ascent vs. descent might be affected by vaccine administration during the outbreak. Tdap vaccination during the 2012 epidemic in Minnesota was examined in the Appendix. Briefly, the largest increase in coverage rates for the 11–14 year olds took place during the outbreak’s descent. Such late coverage should combine with the potential effect of vaccination to contribute to the decline in the proportion of unvaccinated individuals among cases during the outbreak’s descent. No such decline was observed in the data, which in particular suggests no evidence for the effect of vaccination on susceptibility to infection.

This apparent lack of protection from Tdap against infection during an outbreak appears to differ from the estimated effect of DTaP in younger children. A significant reduction in the proportion of cases from the pre-peak to the post-peak period among children who had not received the 5th dose of DTaP, compared to those who had, exhibits a more rapid depletion of the pool of susceptibles among under-vaccinated individuals. This suggests that the 5th dose provides protection from pertussis infection to the 5 year and 8–10 year age groups. No corresponding relative depletion of susceptibles could be detected among under-vaccinated 6–7 year olds; however, the low case counts in the data (a total of seven under-vaccinated cases in that age group) resulted in a wide confidence interval. Our results highlight the importance of achieving high levels of compliance with the pertussis vaccination schedule, as well as timely administration of the fifth dose of DTaP.

Lack of a protective effect from Tdap against pertussis infection, combined with presence of the corresponding effect for DTaP suggests that Tdap vaccination might have a limited role in providing herd immunity and decreasing circulation of *B. pertussis* in the community. A possible factor could be waning of immunity. A recent study estimated the vaccination effectiveness of Tdap against detectable pertussis to drop to 68.2% one year out from receipt, and 34.5% two years out^10^. While it is not unlikely that similar waning would also apply to effectiveness against pertussis infection, our results, particularly for the 11–12 year olds for whom vaccination with Tdap had taken place relatively recently, might point to another factor, namely a more limited effect of Tdap on protection from infection with *B. pertussis* compared to prevention of detectable or symptomatic pertussis episodes that represent the more severe manifestation of an infection.

There are some limitations to this study. We have assumed that the reporting rate within each age group over time and across locations is constant. This assumption seems reasonable as changes in reporting practices through the course of one season are unlikely, save perhaps for the case of young adolescents due to increased awareness among medical staff and the public about the high attack rates in those age groups as the epidemic progressed. Such change would amplify the number of reported cases for those age groups in the later stages of the outbreak, underestimating the RR for age groups that nonetheless were found to carry the highest estimates of RR for that epidemic. Because contact patterns among school-age children are likely to be markedly different during the summer, we repeated the age group analyses, excluding the summer period (weeks 22 to 36) from the pre- and post-peak periods (Appendix). We found that results were similar, but the reduced sample size produced greater uncertainty (Appendix: Table S2).

We have assumed that the epidemic peaks, both overall and age-specific, occur at approximately the same time in all counties. The outbreaks in each of the 19 counties considered in this study occurred at approximately the same time (Appendix: Figure S2), and most counties were clustered around the Twin Cities metropolitan area (Appendix: Figure S1). These counties represented the vast majority of cases in Minnesota, and as such, small, temporally discordant outbreaks in distant rural counties would have little impact on the results. This temporal synchrony suggests in particular that our results on pertussis vaccination should not be sensitive to potential geographic differences in vaccination coverage.

Findings from this study are based on data collected in a single US state, and may not be reflective of pertussis transmission dynamics in the US as a whole. It would be of much interest to repeat this analysis in other US states to determine whether results found in Minnesota are repeated elsewhere. Larger datasets may offer a greater insight into the protective effect of the DTaP and Tdap vaccines. Furthermore, data collected from countries with differing vaccination schedules could provide further information on age group susceptibility and vaccination efficacy.

In conclusion, our analysis provides additional evidence towards the protective effect of the DTaP vaccine. Furthermore, our study highlights the importance of targeting adolescents in infection control measures, and the need for a vaccine providing effective, long-lasting protection against subclinical and symptomatic pertussis infection, and a reduced potential for onward transmission^27^.

## Additional Information

**How to cite this article**: Worby, C. J. *et al.* Examining the role of different age groups, and of vaccination during the 2012 Minnesota pertussis outbreak. *Sci. Rep.* **5**, 13182; doi: 10.1038/srep13182 (2015).

## Supplementary Material

Supplementary Information

## Figures and Tables

**Table 1:** The total number of cases, relative risk (RR) and 95% confidence interval (CI) for nine age groups in the pre- versus post-peak periods during the 2012 Minnesota outbreak.

Age Group (years)	Total Cases		RR(g) (95% CI)
	Pre-peak	Post-peak	
<1	68	56	0.63 (0.44, 0.89)
1–2	92	89	0.53 (0.40, 0.71)
3–4	78	81	0.50 (0.37, 0.67)
5–7	135	103	0.68 (0.53, 0.87)
8–10	380	146	1.35 (1.13, 1.61)
11–12	425	102	2.15 (1.76, 2.63)
13–14	302	100	1.56 (1.26, 1.93)
15–19	248	145	0.88 (0.73, 1.07)
>20	293	244	0.62 (0.53, 0.72)
Total	1984	1025	

**Table 2:**
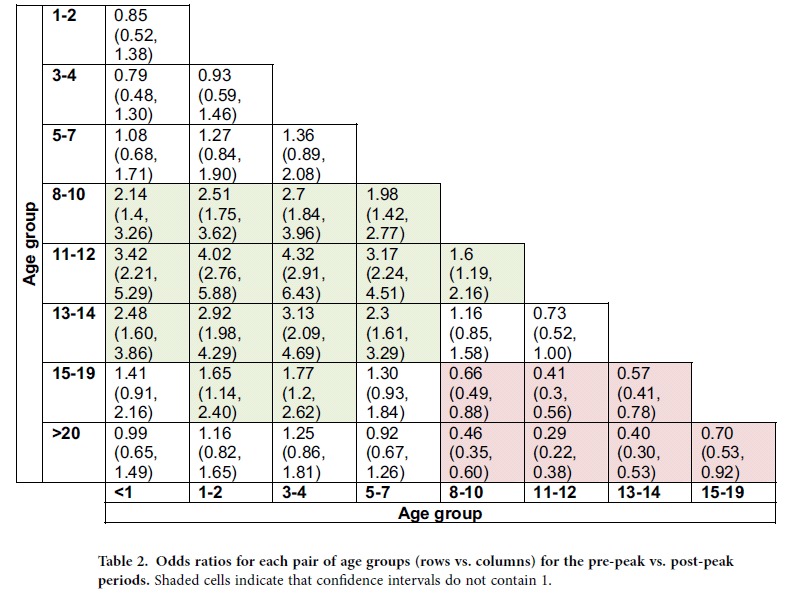
Odds ratios for each pair of age groups (rows vs. columns) for the pre-peak vs. post-peak periods.

Shaded cells indicate that confidence intervals do not contain 1.

**Table 3:** The total cases, odds ratio (OR) and 95% confidence interval (CI) for under-vaccinated vs. fully vaccinated children, pre- and post-peak, during the 2012 Minnesota pertussis outbreak.

Age group	Vaccine status	Total Cases		OR (95% CI)
		Pre-peak	Post-peak	
6–12 months	3 DTaP doses	10	11	0.62 (0.10, 3.53)
	<3 DTaP doses	6	4	
7–14 months	3 DTaP doses	17	16	0.27 (0.005, 3.17)
	<3 DTaP doses	4	1	
15–36 months	4 DTaP doses	36	22	0.96 (0.27, 3.12)
	<4 DTaP doses	12	7	
19–47 months	4 DTaP doses	62	42	0.62 (0.16, 2.06)
	<4 DTaP doses	12	5	
5 years	5 DTaP doses	18	20	0.16 (0.01, 0.84)
	<5 DTaP doses	12	2	
6–7 years	5 DTaP doses	92	38	0.97 (0.09, 6.24)
	<5 DTaP doses	5	2	
8–10 years	5 DTaP doses	342	97	0.13 (0.003, 0.82)
	<5 DTaP doses	27	1	

**Table 4:** The total cases, odds ratio (OR) and 95% confidence interval (CI) for children who lacked vs. had Tdap vaccination, pre- and post-peak, during the 2012 Minnesota pertussis outbreak.

Age group	Subpopulations	Total Cases		OR (95% CI)
		Pre-peak	Post-peak	
11–12	Tdap	90	13	1.06 (0.53, 2.25)
	No Tdap	281	43	
13–14	Tdap	237	60	1.23 (0.34, 3.71)
	No Tdap	16	5	
