# Supplementary material for: Examining the role of different age groups, and of vaccination during the 2012 Minnesota pertussis outbreak
Source: Sci Rep. 2015 Aug 17;5:13182. doi: 10.1038/srep13182 (PMC4538373; doi:10.1038/srep13182)
Supplement: Supplementary Information [file srep13182-s1.doc]

Supporting Information for “Examining the role of different age groups, and of vaccination during the 2012 Minnesota pertussis epidemic”

Colin J. Worby, Cynthia Kenyon, Ruth Lynfield, Marc Lipsitch, Edward Goldstein

**1. County level data**

In this section we present additional data on the 19 counties used in the main analysis: their geographic location (Figure S1), annual number of cases between 2010-2013 (Table S2) and the weekly incidence between 2010-2013 (Figure S2)

**Figure S1**: The 19 Minnesota counties used in the main analysis. Counties not included in our study are shaded in gray. This map was created in R using the ‘maps’ package [8].

|  | **Reported cases by year** | | | |
| --- | --- | --- | --- | --- |
| **County** | **2010** | **2011** | **2012** | **2013*** |
| Hennepin | 233 | 169 | 970 | 156 |
| Sherburne | 33 | 10 | 102 | 14 |
| Carver | 22 | 15 | 89 | 19 |
| Dakota | 130 | 55 | 277 | 65 |
| Stearns | 74 | 44 | 131 | 44 |
| Wright | 97 | 35 | 274 | 9 |
| Anoka | 44 | 34 | 498 | 24 |
| Crow Wing | 6 | 6 | 54 | 1 |
| Ramsey | 91 | 56 | 234 | 173 |
| Olmstead | 28 | 19 | 238 | 40 |
| Washington | 50 | 22 | 260 | 41 |
| Scott | 16 | 9 | 177 | 18 |
| Isanti | 6 | 4 | 47 | 0 |
| St. Louis | 19 | 31 | 81 | 25 |
| Chisago | 4 | 3 | 76 | 7 |
| Winona | 0 | 28 | 75 | 23 |
| Itasca | 2 | 2 | 48 | 2 |
| Freeborn | 10 | 0 | 88 | 0 |
| Mower | 0 | 2 | 43 | 15 |
| Minnesota total † | 1143 | 661 | 4144 | 1034 |

† http://www.health.state.mn.us/divs/idepc/diseases/pertussis/stats/

*provisional

**Table S1:** Annual numbers of cases in the 19 counties used in the main analysis

**Figure S2:** Weekly recorded pertussis incidence between 2010-2013 in the 19 counties used in the main analysis

**2. More on the interpretation of RR and the odds ratio for vaccinated vs. unvaccinated cases**

The definition of RR(g) in the main body of the text refers to the ratio of the proportions of each age group among detected pertussis cases for periods before vs. after the peak of an epidemic. Since case-detection ratios may vary substantially by age, proportion among detected cases can be though of as a proportion among weighted incidence. To exhibit a more clear relation of RR to incidence that is independent of weighting, we examine the ratio $RR(g_{1})/RR(g_{2})$ of RRs in two age groups (say $g_{1}$ and $g_{2}$). To fix notation, let $CB_{i}$ and $CA_{i}$be the number of detected pertussis cases before and after the peak in group $g_{i}$, and let $CB_{t}$ and $CA_{t}$ be the total number of detected cases before and after the peak respectively. Then

$$\frac{RR(g_{1})}{RR(g_{2})}=(\frac{CB_{1}}{CB_{t}}/\frac{CA_{1}}{CA_{t}})/(\frac{CB_{2}}{CB_{t}}/\frac{CA_{2}}{CA_{t}})=\frac{CB_{1}}{CA_{1}}/\frac{CB_{2}}{CA_{2}}$$

If case-detection rates in each age group remain constant over the course of the epidemic (or, at least those rates change by the same factor over time for all age groups), $RR(g_{1})/RR(g_{2})$ can be related to incidence $IB_{i}$ and $IA_{i}$ before and after the peak in groups $g_{1}$ and $g_{2}$:

$\frac{RR_{1}}{RR_{2}}=\frac{IB_{1}}{IA_{1}}/\frac{IB_{2}}{IA_{2}}=\frac{IB_{1}}{IB_{2}}/\frac{IA_{1}}{IA_{2}}$= $OR(g_{1},g_{2})$ (S1)

with the last expression representing the odds ratio $OR(g_{1},g_{2})$ for being in group $g_{1}$ vs. group $g_{2}$ for the incident cases during the before-the-peak period relative to the after-the-peak period.

This same argument applies to the odds ratio of detected unvaccinated and vaccinated cases during the before vs. after the peak periods. Let $IB_{V}$ and $IB_{UV}$ is the incidence of vaccinated and unvaccinated cases before the peak, and $IA_{V}$ and $IA_{UV}$ be the corresponding incidences after the peak. Let $CB_{V},CB_{UV},CA_{V},CA_{UV}$ be the corresponding counts for the detected cases. Then the odds ratio

$$\frac{CB_{UV}}{CB_{V}}/\frac{CA_{UV}}{CA_{V}}=\frac{CB_{UV}}{CA_{UV}}/\frac{CB_{V}}{CA_{V}}=\frac{IB_{UV}}{IA_{UV}}/\frac{IB_{V}}{IA_{V}}=\frac{IB_{UV}}{IB_{V}}/\frac{IA_{UV}}{IA_{V}}$$

which is the odds ratio of the incidences of vaccinated and unvaccinated cases before vs. after the peak under the same assumption of constant reporting rates for each category of vaccinated and unvaccinated individuals.

**3. Credible intervals for RR(g)**

We follow the inference framework in <http://sphweb.bumc.bu.edu/otlt/MPH-Modules/BS/BS704_Confidence_Intervals/BS704_Confidence_Intervals8.html>. Let $RR'=\frac{CB_{g}}{CB}/\frac{CA_{g}}{CA}$ be the point estimate of $RR(g)$. To produce confidence bounds on this estimate we note that the distribution of $\ln(RR(g))$ is approximately normal. The confidence bounds for $\ln(RR(g))$ are

$$\ln(RR')\mp1.96\sqrt{\frac{(CB-CB_{g})}{CB_{g}\cdot CB}+\frac{(CA-CA_{g})}{CA_{g}\cdot CA}}$$

Exponentiating the above produces confidence bounds for $RR(g)$, as shown in Table 1 in the main text.

**4. Excluding the summer weeks**

We interpreted the estimates of RR(g) for the different population groups as being indicative of the relative depletion of susceptible individuals in those age groups during the epidemic’s ascent. However changes in RR may also occur due to changes in mixing patterns, particularly during the summer school vacation period that may result in changes in the relative roles of the different age groups in pertussis incidence. To address this, we performed analysis similar to the one in the main body of the text for the estimation of RR(g), with the summer weeks (weeks 22-36, 2012) excluded from the before- and after- the peak periods. Table S2 records the results of this analysis.

| **Age Group** | **RR(g) (95% CI)** |
| --- | --- |
| <1 | 0.66 (0.41, 1.07) |
| 1-2 | 0.62 (0.33, 1.08) |
| 3-4 | 0.59 (0.34, 0.96) |
| 5-7 | 0.75 (0.50, 1.10) |
| 8-10 | 1.31 (1.01, 1.72) |
| 11-12 | 2.09 (1.54, 2.85) |
| 13-14 | 1.75 (1.27, 2.43) |
| 15-19 | 0.71 (0.55, 0.92) |
| >20 | 0.61 (0.49, 0.75) |

**Table S2:** The relative risk (RR) and 95% credible interval (CI) for several age groups in the pre- versus post-peak periods during the 2012 Minnesota outbreak, excluding summer weeks (weeks 22-36).

We see that the results in Table S3 are quite similar to ones in Table 1, with the highest estimate of RR belonging to the 11-12 year olds, followed by the 13-14 and the 9-10 year olds. Moreover the estimates of RR(g) are below 1 for all the other age groups in both Tables 1 and S3.

**5. Exploring the relation between RR and transmission dynamics by simulations**

In this section we exhibit some basic relations between transmission dynamics and RR(g) in various age groups by simulations and show how changes in susceptibility in some age groups (e.g. as a result of vaccination) translate into corresponding changes in the RR(g)s. In the next section, similar issues are addressed more systematically in an analytical framework.

We adopt the stratified mass action transmission model [1,2]. A key ingredient in this framework is the contact matrix reflecting contact patterns between the different age groups. For illustration purposes, we utilize the POLYMOD data from [4]. That data allows one to consider the following 15 age groups: (0-4,5-9,..,60-64,65-69,70+). Contact matrices for those age groups estimated for the different European countries [4] are averaged to obtain the contact matrix we use in our simulations.

Another key set of parameters affecting the relative role of the different age groups is group-specific relative susceptibilities and infectivities. Previous analyses suggest that the latter are group-independent while the former vary a great deal with age, at least for influenza [3]. We adopt the same assumption here, setting all age groups equally infectious. For the relative susceptibilities, we consider the following two scenarios:

***Baseline scenario:*** To broadly mimic the estimates in Tables 1 and S2, we assume that the relative susceptibility is 0.5 for those aged 15+ (due to receipt of whole-cell vaccine, etc.), as well as for those aged 0-4 (due to the frequency of DTaP vaccination among the young). The relative susceptibility for 5-9 and 10-14 year olds is assumed to equal 1. Figure S3 exhibits the results of the transmission dynamics under the above assumptions, the (time-varying) proportions of each age group in the overall incidence, and the values for RR(g) in the various age groups.

We see that children 10-14 have by far the highest relative proportion in incidence during the outbreak’s ascent (and the highest estimate of RR), followed by children 5-9, who are assumed to have the same susceptibility as the 10-14 years olds, but less contacts in the POLYMOD data [4]. Children 15-19 have the 3^rd^ largest estimate of RR; while the have more contacts than 5-9 year olds in the POLYMOD data, the fact that their relative susceptibility if half the one of 5-9 year olds tips the balance in terms of the relative role in incidence during the outbreak’s ascent in favor of the 5-9 year olds.

**Figure S3:** Incidence curve (black), time-varying proportions of the different age groups in the overall incidence, and the RR values for those age groups under the baseline scenario.

***Reduced susceptibility of the 10-14 year olds scenario:*** We make the same assumptions as before but reduce the relative susceptibility among the 10-14 year olds to 0.7. Figure S4 plots the corresponding results. We see a drastic reduction in the role of 10-14 year olds in incidence during the outbreak’s ascent, with 5-9 year olds surpassing them in the proportion among the overall incidence as well as the estimate of RR.

**Figure S4:** Incidence curve (black), time-varying proportions of the different age groups in the overall incidence, and the RR values for those age groups under the reduced susceptibility of the 10-14 year olds scenario.

**6. Relation between depletion of susceptibles in a population group and the potential impact of vaccination on the epidemic’s reproductive number**

In this section we examine the relation between the rate of depletion of susceptibles in a population group $i$ (which is related to RR(i)) and the impact of vaccinating group $i$ on the epidemic’s reproductive number. Unlike the previous section, that was more exploratory and meant to illustrate the basic relation between RR and transmission dynamics by a few simulations, this one addresses the issue more systematically in the stratified mass action framework.

We follow the stratified mass action transmission-modeling framework ([1,2]). In this framework, the population is divided into strata $i=1,..,N$ where each stratum $i$ has $n_{i}$ individuals of which $s_{i}$ are initially susceptible, each having susceptibility $a_{i}$ and infectivity $c_{i}$ upon infection (the latter are usually deemed stratum-independent, at least for influenza [3,4]). Moreover the contact rate between average individuals in strata $i$ and $j$ is $b_{ij}$ which is symmetric in $i$ and $j$ (with estimates for such rates existing in the literature [5,6]). The next generation matrix $K(t)=(K_{ij}(t))$ equaling the average number of infections in stratum $i$ caused by an individual in stratum $j$ who got infected at time $t$ can be estimated as

$K_{ij}(t)=\lambda\cdot s_{i}(t)\cdot a_{i}\cdot b_{ij}\cdot c_{j}$ (1)

(here $t=0$ corresponds to the initial, exponential growth phase of the epidemic which we shall consider henceforth, with $s_{i}(0)=s_{i}$).

The effective reproductive number $R(t)$ at time t is the leading eigenvalue of K, with the leading eigenvector of being estimated as the incidence vector $x(t)=(x_{1}(t),..,x_{N}(t))$. Equation (1) in [1] is a formula for the derivative of the logarithm of the reproductive number with respect to vaccination of different subgroups, where $q_{i}$ is the vaccine efficacy in group $i$. The model in [1,2] uses a 2-parameter representation of susceptibility in the different population groups, namely a group $i$ has $s_{i}$ initially susceptible individuals, each having susceptibility $a_{i}$. Let the *average initial* susceptibility in group $i$ be

$d_{i}=\frac{a_{i}s_{i}}{n_{i}}$ (2)

Since the infectivities and susceptibilities are defined up to common joint scaling (in opposite directions), we can always assume that

$d_{i}\leq1$ (3)

We note that the initial next generation matrix K depends only on $d_{i}$ (and $n_{i}$, eq. 1), and so does its leading eigenvector $x$. Unsurprisingly, the right hand side in eq. 1 in [1] in the beginning of an outbreak also only depends on $d_{i}$. However with the matrices D, B, C fixed, the initial *rate of depletion of susceptibles* = *force of infection*

$F_{i}=\frac{x_{i}}{s_{i}}$ (4)

does depend on $s_{i}$. Thus we can scale down $s_{i}$ in a given population group $i$ (and scale up $a_{i}$ correspondingly) leaving the initial impact of vaccination on the reproductive number in all population groups fixed to make the initial rate of depletion of susceptibles (and RR) in group $i$ arbitrary large. However such arbitrary upward scaling of $a_{i}$ appears implausible biologically. We propose to consider a class of models for which initial relative depletion of susceptibles (eq. 4) can in fact be directly related to the impact of vaccination on the reproductive number. In this class of models, which we call *balanced*, we assume that

$s_{i}=a_{i}n_{i}$ (5)

In other words, if a group is ½ susceptible, it doesn’t mean that half the individuals are fully susceptible, or everyone is half susceptible, but $\frac{1}{\sqrt{2}}$ individuals are $\frac{1}{\sqrt{2}}$ susceptible. More generally, the solution to eqns. 5 and 2 is

$s_{i}=\sqrt{d_{i}}\cdot n_{i}$ , $a_{i}=\sqrt{d_{i}}$

which is always possible ($s_{i}$ is indeed smaller than $n_{i}$) due to eq. 3. In this class of models, if we assume that infectivities ([3,4]) and vaccine efficacies are group independent, we could re-write eq. 1 in [1] as

$\frac{d(\ln(R))}{du_{i}}=-h'(F_{i})^{2}$ (6)

Thus in this class of models vaccinating the group with the highest *initial rate of depletion of susceptibles* would also have the highest *initial impact on the reproductive number*.

**7. Tdap vaccination during the outbreak**

Figure S5 presents the weekly Tdap vaccination rates among the 11, 12, 13 and 14 year olds in Minnesota, as well as vaccination coverage rates in those age groups during certain time points in 2012.


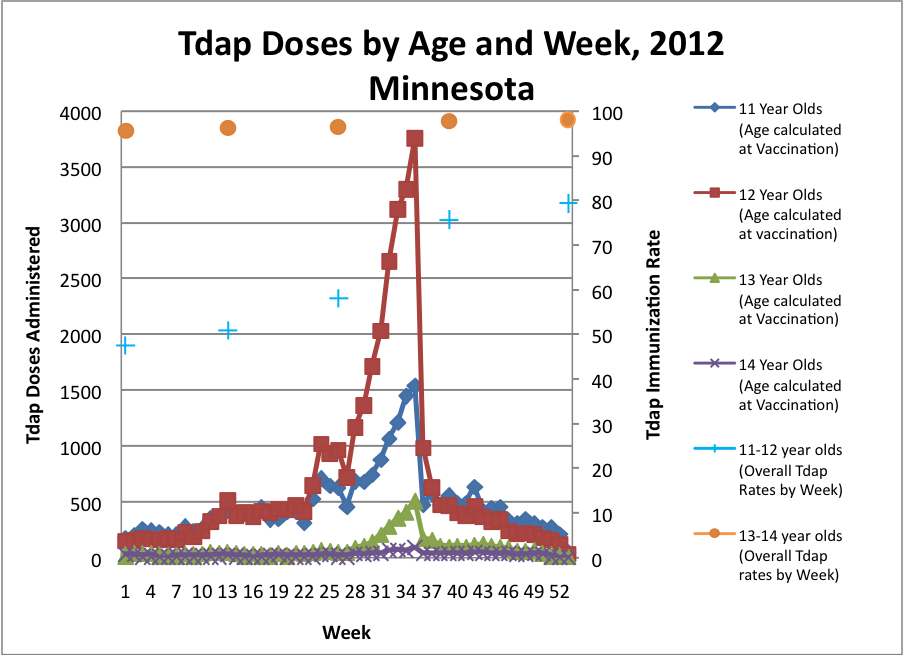


**Figure S5:** Weekly Tdap vaccination rates among the 11, 12, 13 and 14 year olds, as well as vaccination coverage rates in 11-12 and 13-14 year olds at certain time points in 2012. Data provided by the Minnesota Immunization Information Connection (MIIC).

We see that a larger increase in coverage rates and, correspondingly, an even larger relative decline in the proportion of adolescents who are unvaccinated took place in the after-the-peak period (weeks 31-42) compared with the before-the-peak period (prior to week 27). As mentioned in the Discussion, such late coverage should combine with the potential effect of vaccination to contribute to the decline in the proportion of unvaccinated individuals among cases during the outbreak’s descent, a decline that was not observed in the data.

8. **Investigating the relation between RR and driver role during epidemics in a simulation framework**

In section 6 of the Supporting Information we investigated the relation between the initial rate of the depletion of susceptibles and the impact of vaccination in a given age group on the epidemic’s reproductive number. Here, we explore the relation between the summary statistic RR and the effect of vaccination in a given age group on the epidemic’s reproductive number. We note that administration of a perfect vaccine amounts to removal of an individual from transmission dynamics – thus a group for which vaccination has the highest impact on the reproductive number can be also thought of as the group for which the average member has the largest impact on propagating (driving) the epidemic.

We adopt the modeling framework of section 6. We split the population into the following ten age groups (<1,1-3,4-6,7-10,11-14,15-18,19-29,30-49,50-64,65+). Population size in each group in Minnesota is obtained from [9]. Based on previous work on the serial interval of pertussis [10,11], we assume that each infected individual is infectious for a mean of 24 days. The contact matrix $(b_{ij})$ is obtained from the POLYMOD study data [12], averaging out the estimates for eight European countries. We assume that infectivity is group-independent. Given the uncertainty about the initial susceptibility in different population groups, we assume that the average initial susceptibility (eq. 2) in each group is either 0.1 or 0.05 – thus either 31.6% of individuals have susceptibility 0.316 (the rest are not susceptible), or 22.4% of individuals have susceptibility 0.224. Altogether, $2^{10}=1024$ scenarios are considered.

We choose the number $\lambda$ in eq. 1 so that in the maximal susceptibility scenario (each group having average susceptibility 0.1), the epidemic’s initial reproductive number is 1.3 (maximal possible). For the 1,024 epidemics considered, those that don’t take off (initial reproductive number below 1) are discarded. For the remainder of the epidemics, we compute, for each age group, its RR and the impact of administering vaccine quantity equaling 1% of the Minnesota population to that age group on the epidemic’s reproductive number. Our objective is to examine whether the group with the highest RR is also the group for which vaccine administration has the largest impact on reducing the epidemic’s initial reproductive number.

A total of 643 outbreaks were successful out of the 1024 scenarios considered. Among the successful outbreaks, 11-14 year olds had the highest RR in 74% of scenarios, while RR was highest for 15-18 year olds in 14% of cases. In 83% of cases, the age group with the largest RR was also the group for which administering vaccination as described above would have the largest impact. In each of the 250 simulations with the highest initial reproductive number, 11-14 year olds had the highest RR, and were also the group for which vaccination would have the largest impact. Figure S6 provides a summary of outcomes for 64 scenarios.

**Figure S6.** Simulation outcomes for 64 scenarios. With susceptibility for all adult age groups equal to 0.1, simulation results for the 64 permutations of high/low susceptibility for the six groups aged 18 and younger are shown here. Each box represents a scenario, with age group susceptibility levels indicated on the axes. The color of the box denotes the initial reproductive number for the given scenario, while points indicate whether the age group with the highest RR was also the group for which vaccination would have the largest impact. Boxes without points represent unsuccessful outbreaks, with an initial R0<1.

SUPPLEMENTARY REFERENCES

[1] Wallinga J, van Boven M, Lipsitch M. Optimizing infectious disease interventions during an emerging epidemic. PNAS 2010;17(2) 923-928

[2] [Goldstein E](http://www.ncbi.nlm.nih.gov/pubmed?term="Goldstein E"%5BAuthor%5D&itool=EntrezSystem2.PEntrez.Pubmed.Pubmed_ResultsPanel.Pubmed_RVAbstract), [Apolloni A](http://www.ncbi.nlm.nih.gov/pubmed?term="Apolloni A"%5BAuthor%5D&itool=EntrezSystem2.PEntrez.Pubmed.Pubmed_ResultsPanel.Pubmed_RVAbstract), [Lewis B](http://www.ncbi.nlm.nih.gov/pubmed?term="Lewis B"%5BAuthor%5D&itool=EntrezSystem2.PEntrez.Pubmed.Pubmed_ResultsPanel.Pubmed_RVAbstract), [Miller JC](http://www.ncbi.nlm.nih.gov/pubmed?term="Miller JC"%5BAuthor%5D&itool=EntrezSystem2.PEntrez.Pubmed.Pubmed_ResultsPanel.Pubmed_RVAbstract), [Macauley M](http://www.ncbi.nlm.nih.gov/pubmed?term="Macauley M"%5BAuthor%5D&itool=EntrezSystem2.PEntrez.Pubmed.Pubmed_ResultsPanel.Pubmed_RVAbstract), [Eubank S](http://www.ncbi.nlm.nih.gov/pubmed?term="Eubank S"%5BAuthor%5D&itool=EntrezSystem2.PEntrez.Pubmed.Pubmed_ResultsPanel.Pubmed_RVAbstract), [Lipsitch M](http://www.ncbi.nlm.nih.gov/pubmed?term="Lipsitch M"%5BAuthor%5D&itool=EntrezSystem2.PEntrez.Pubmed.Pubmed_ResultsPanel.Pubmed_RVAbstract), [Wallinga J](http://www.ncbi.nlm.nih.gov/pubmed?term="Wallinga J"%5BAuthor%5D&itool=EntrezSystem2.PEntrez.Pubmed.Pubmed_ResultsPanel.Pubmed_RVAbstract). Distribution of vaccine/antivirals and the 'least spread line' in a stratified population. [J R Soc Interface.](javascript:AL_get(this, 'jour', 'J R Soc Interface.');) 2010 May 6;7(46):755-64

[3] Rohani P, Zhong X, King A. Contact network structure explains the changing epidemiology of pertussis. Science 2010;330(6006) 982-985

[4] Cauchemez S, Donnely C, Reed C, et al. Household Transmission of 2009 Pandemic Influenza A (H1N1) Virus in the United States. N Engl J Med 2009; 361:2619-2627

[5] Mossong J, Hens N, Jit M, et al. Social contacts and mixing patterns relevant to the spread of infectious diseases. PLoS medicine. 2008;5(3):e74

[6] Wallinga J, Teunis P, Kretzschmar M. Using data on social contacts to estimate age-specific transmission parameters for respiratory-spread infectious agents. Am J Epidemiol. 2006;164(10):936-944.

[7] Ross SM. A first course in probability. 8th ed. Upper Saddle River, N.J.: Pearson Prentice Hall; 2010. xiii, 530 p. p.

[8] Original S code by Richard A. Becker and Allan R. Wilks. R version by Ray Brownrigg. Enhancements by Thomas P Minka (2014). maps: Draw Geographical Maps. R package version 2.3-9. <http://CRAN.R-project.org/package=maps>

[9] CDC Wonder. Bridged-Race Population Estimates 1990-2013. <http://wonder.cdc.gov/Bridged-Race-v2013.HTML>

[10] Vink MA, Bootsma MC, Wallinga J. Serial intervals of respiratory infectious diseases: a systematic review and analysis. Am J Epidemiol. 2014 Nov 1;180(9):865-75

[11] Te Beest DE, Henderson D, van der Maas NA, de Greeff SC, Wallinga J, Mooi FR, van Boven M. Estimation of the serial interval of pertussis in Dutch households. Epidemics 2014; 7:1-6

[12] J. Mossong, N. Hens, M. Jit, P. Beutels, K. Auranen, et al. Social contacts and mixing patterns relevant to the spread of infectious diseases PLoS Med., 5 (2008), p. e74
